# Effects of dezocine on morphine tolerance and opioid receptor expression in a rat model of bone cancer pain

**DOI:** 10.1186/s12885-021-08850-0

**Published:** 2021-10-20

**Authors:** Lin-xin Wu, Yan-peng Dong, Qian-mei Zhu, Bo Zhang, Bo-lun Ai, Tao Yan, Guo-hua Zhang, Li Sun

**Affiliations:** 1 Department of Anesthesiology, National Cancer Center/National Clinical Research Center for Cancer/Cancer Hospital, Chinese Academy of Medical Sciences and Peking Union Medical College, Beijing, 100021 China; 2 Department of Anesthesiology, National Cancer Center/National Clinical Research Center for Cancer/Cancer Hospital & Shenzhen Hospital, Chinese Academy of Medical Sciences and Peking Union Medical College, Shenzhen, 518100 China; 3 Department of Breast Surgical Oncology, National Cancer Center/National Clinical Research Center Cancer/Cancer Hospital, Chinese Academy of Medical Sciences and Peking Union Medical College, Beijing, 100021 China

**Keywords:** Dezocine, Tolerance, Morphine, Opioid receptors, Bone cancer pain

## Abstract

**Background:**

Clinically, the coadministration of opioids to enhance antinociception and decrease tolerance has attracted increasing research attention. We investigated the effects of dezocine, a mu- and kappa-opioid receptor agonist/antagonist, on morphine tolerance and explored the involvement of opioid receptor expression in a rat model of bone cancer pain.

**Methods:**

Thermal nociceptive thresholds were measured after the subcutaneous injection of morphine (10 mg/kg) alone or combined with dezocine (10 or 1 mg/kg) for 7 consecutive days. Real-time PCR and western blot analysis were used to examine opioid receptor expression in the periaqueductal gray (PAG) and spinal cord.

**Results:**

The analgesic effect was significantly decreased after 4 days of morphine administration. We observed that low-dose dezocine significantly attenuated morphine tolerance without reducing the analgesic effect of morphine. Low-dose dezocine coadministration significantly reversed the downregulated expression of mu (MOR) and delta (DOR) opioid receptors in the PAG and the upregulated expression of kappa (KOR) and DOR in the spinal cord induced by morphine. Moreover, low-dose dezocine coadministered with morphine significantly inhibited KOR expression in both the PAG and spinal cord.

**Conclusions:**

The combination of low-dose dezocine with morphine may prevent or delay the development of morphine tolerance in a rat model of bone cancer pain. The regulation of opioid receptor expression in the PAG and spinal cord may be part of the mechanism.

## Introduction

Cancer pain is a common symptom in cancer patients, and 70% of patients with advanced cancer can develop symptoms of pain [1]. Despite the availability of effective treatments, cancer-related pain may be inadequately controlled in up to 50% of patients. Opioids are the most important drugs to treat moderate to severe cancer pain.

Clinically, opioid coadministration to enhance antinociception and decrease tolerance has increasingly attracted the attention of clinicians and researchers [2, 3]. Recent studies have demonstrated that a combination of morphine and low-dose opioid receptor agonists/antagonists has good analgesic effects and causes fewer adverse reactions than morphine alone [4, 5]. Opioid receptor agonists-antagonists, including dezocine, pentazocine and buprenorphine, are a class of opioid drugs that are widely used in clinical anesthesia and pain therapy. Although the combination of morphine with opioid receptor agonist antagonists is commonly used in clinical practice, there is still controversy over its effect [6, 7]. Yekkirala et al. suggested that when the opioid receptor agonist-antagonist pentazocine was combined with morphine, pentazocine antagonized morphine-induced activation of μ receptors, thus weakening its analgesic effect [8]. In contrast, Aurilio et al. found that opioid receptor agonist-antagonist buprenorphine and morphine have a synergistc analgesic effect [9]. In addition, butorphanol in combination with morphine can reduce the incidence of morphine-induced pruritus [10].

Dezocine is a representative opioid receptor agonist/antagonist that is widely used in clinical practice in China for treating postoperative and even cancer pain [11]. When combined with fentanyl, dezocine could reduce the incidence of cough caused by intravenous fentanyl [9]. However, there have been few reports on the analgesic effect and analgesic tolerance of dezocine alone or in combination with opioid agents in the treatment of cancer pain. Some parts of brain are very important regions for pain perception, transmission and modulation such as PAG, locus coeruleus, amygdala and spinal cord. Opioid receptors were mainly found in PAG, thalamus, locus coeruleus and spinal cord. In the mechanism research of analgesic effects and tolerance of opioid drugs, it is still very important to study opioid receptors in various parts of brain, including the types and expression of receptors, interactions between receptors and receptor genes [12–14]*.* The present study investigated the analgesic effect and tolerance of dezocine in combination with morphine in a rat bone cancer pain (BCP) model and detected the mRNA and protein expression of opioid receptors in PAG and spinal cord which may induced by morphine and/or dezocine, aiming to provide preliminary data on cotreatment therapy and opioid receptor-related mechanisms of cancer pain.

## Materials and methods

### Animals

A total of 70 female Wistar rats weighing 160 to 200 g were used for this study. Animals were obtained from Vital River Laboratories (Beijing, China). All rats were reared under a 12 h light/dark schedule at room temperature (22 ± 2 °C) and a humidity of 40–50%. Food and water were supplied ad libitum. These animal experiments were approved by the ethics committee for animal care of the Cancer Hospital, Chinese Academy of Medical Sciences (No. NCC2013A057), and followed the National Institutes of Health Guidelines for the Care and Use of Laboratory Animals.

### Cell line

Half a milliliter of Walker 256 rat breast cancer cells (2 × 10^7^ cells/mL) was intraperitoneally injected into Wistar rats. One week later, 3 mL of ascites was collected and diluted with normal saline to prepare a cell suspension at a density of 2 × 10^7^ cells/mL for injection.

### Rat model of tibial bone cancer pain

A rat model of BCP was established following a previous report [15]. Rats in the BCP group were anesthetized with pentobarbital (65 mg/kg) and tied in the supine position. A small incision was made in the lower 1/3 of the left tibia. After incision of the skin and separation of the muscle to expose the lower 1/3 of the anteromedial tibia, a 10-ml syringe needle was used to drill a hole into the bone marrow cavity. After insertion of the needle 1 cm into the cavity, 10 μl of prepared Walker 256 cell suspension was injected. After injection, the needle was left in the bone marrow cavity for 2 min and then pulled out. The hole was closed with bone wax, the wound was cleaned and sutured, and the rats were placed back in the cages.

### Behavior test of nociception

On days 1, 3, 5, 7, 9, 11, 13, and 15 after surgery, rats were placed on a test bench and allowed to freely walk for 2 min. Flinches (defense time) were defined as holding of the hindpaw aloft while not ambulatory. Normally, the time was 0–2 s. The increase in the time to 8–16 s suggested that the model was successfully developed and could be used for experiments [10, 16].

### Groups and dosing regimens

Fifteen days after bone cancer pain was induced, 36 rats of 54 rats were successfully induced and randomly divided into six groups (*n* = 6 for each) according to the random number table method. Morphine and/or dezocine or saline were subcutaneously given twice daily (at 7:30 am and 7:30 pm) for seven consecutive days. The groups were: as follows morphine group (M group; 10 mg/kg morphine), two dezocine groups (D1 group: 10 mg/kg dezocine; D2 group: 1 mg/kg dezocine), two morphine + dezocine groups (MD1 group: 10 mg/kg morphine + 10 mg/kg dezocine; MD2 group: 10 mg/kg morphine + 1 mg/kg dezocine), and a control group (1 mL isotonic saline).

### Thermal hyperalgesia measurement

The hot plate test was used to measure the thermal nociception of rats with a hotplate apparatus (YLS-6B, ZS Dichuang, China). The test was performed 30 min after drug administration every day. Temperature was maintained at 50 ± 0.5 °C, and paw withdrawal latency (PWL) was recorded with a cutoff time of 30 s to avoid tissue damage. Three measurements were performed in each animal, and the average value was calculated. The interval between each measurement was no less than 5 min. If the thermal nociceptive threshold of the morphine group was significantly different from that of the control group, analgesic tolerance to morphine was established in rats.

### Histology

On day 8 after drug administration, the rats in each group were killed by CO_2_ asphyxiation, and left tibial bone tumor samples were taken, fixed in 4% paraformaldehyde and subjected to pathological examination. Then, the bones were embedded in paraffin, and sections were cut into 4 μm sections (RM2016, Leica, Germany) and stained using the standard hematoxylin and eosin (H&E) method to visualize tumor cell infiltration and bone destruction in BCP rats under a microscope (Nikon Eclipse TI-SR, Leica, Japan).

### Real-time PCR analysis of opioid receptor (OR) mRNA expression

RNA was isolated from periaqueductal gray (PAG) and spinal cord tissues using TRIzol reagent (Gibco Life Technologies, USA). cDNA was synthesized using a PrimeScript RT reagent kit (TaKaRa Biotechnology, Japan). The amplification conditions were set as follows: after 40 cycles of predenaturation at 95 °C for 10 min, denaturation at 95 °C for 30 s, annealing at 60 °C for 30 s, and extension at 72 °C for 40 s, the dissolution curve was determined, and the temperature was increased from 55 °C to 95 °C. The primers were purchased from Qiagen. GAPDH primer: Gapdh F, 5′-GACATCAAGAAGGTGGTGAAGC-3′, Gapdh R, 5′-TGTCATTGAGAGCAATGCCAGC-3′); mu opioid receptor (MOR) primer: Oprm1 F, 5′-AATCGTCAACGTCTGCAACTGG-3′, Oprm1 R, 5′- GAACGTGAGGGTGCAATCTATGG-3′); kappa opioid receptor (KOR) primer: Oprk1 F, 5′-CGAGTAGCATGTACCTTCACTGAG-3′, Oprk1 R, 5′-GTTCAGGAACTGCTTTGTCCAC-3′); delta opioid receptor (DOR) primer: Oprd1 F, 5′-CAACGTGCTCGTCATGTTTGG-3′, Oprd1 R, 5′-CAGGTACTTGGCGCTCTGGAA-3′). The mRNA expression was normalized to that of GAPDH (endogenous control) through the 2^−△△CT^ method.

### Western blotting analysis for OR protein expression

For extraction of total proteins, tissues were homogenized in cold RIPA buffer. The homogenates were centrifuged at 12000×g for 15 min at 4 °C. As previously described [17], protein extracts (50 μg per sample) separated by 7.5% SDS-PAGE were transferred to PVDF membranes. The membranes were blocked using 5% skim milk in Tris-buffered saline with Tween-20 (TBST). After 2 h, the membranes were incubated with the primary antibody at a dilution of 1:1000 in TBS-T overnight at 4 °C (MOR, NB100–1620; DOR, NBP1–19504 and KOR, NB100–91902, Novusbio, USA). Subsequently, membranes were incubated for 2 h with HRP-conjugated secondary antibody. The chemiluminescence method was as follows: ECL hypersensitive luminescent solutions A and B were used, and A and B were mixed in the same volume, incubated with the membrane, and reacted in the dark for 1 min. Quantification analysis of the blots was performed using ImageJ software. Targeted bands were normalized to that of β-actin (1:10000, Santa Cruz Biotechnology, Argentina).

### Statistical analysis

Data are expressed as the mean ± standard error of the mean. Statistical analysis was performed using two-way or one-way analysis of variance with the Bonferroni test for post hoc analysis. The significance level was set at 0.05, and calculations were performed with the aid of SPSS 11.0 software.

## Results

### Bone cancer pain model

**Fig. 1:**
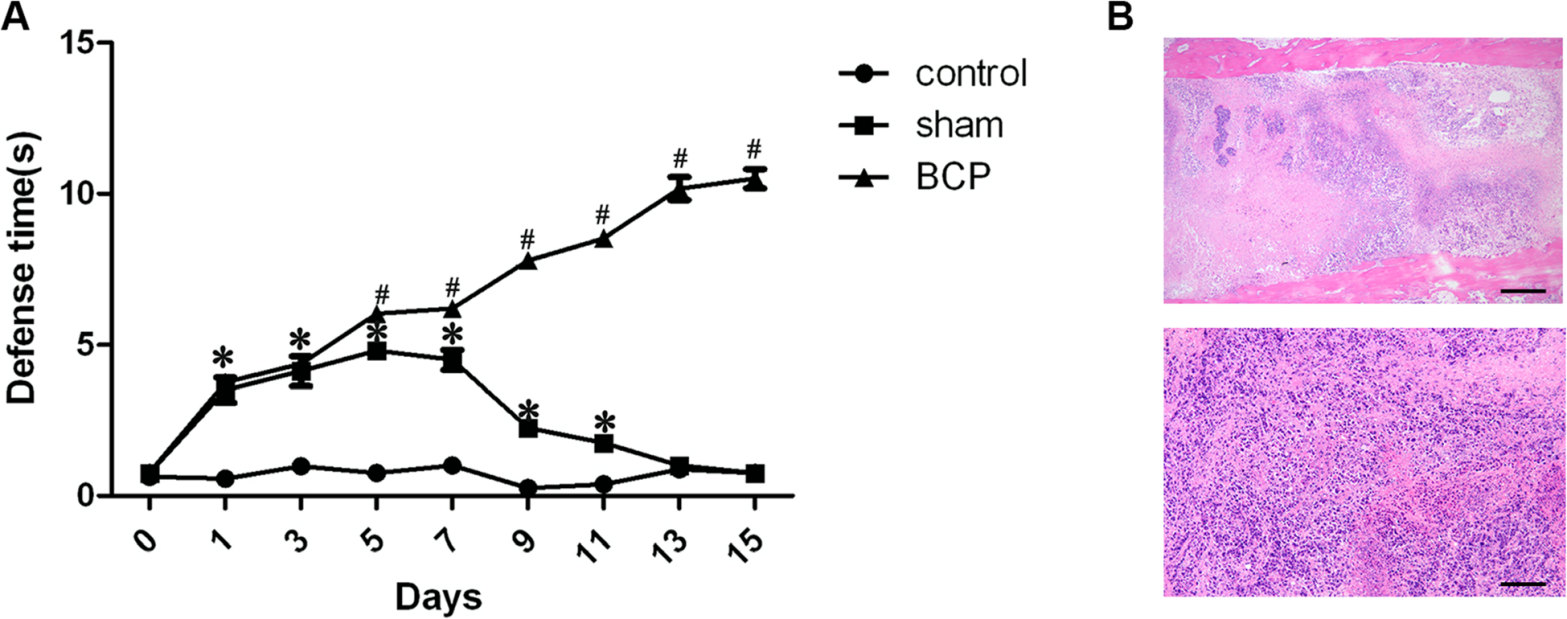
Establishment of a bone cancer pain model. A. Behavioral tests of nociception in the groups. Defense times were measured in three groups: the control group (*n* = 8; skin incision only), sham group (n = 8; intratibial injection of normal saline only), and BCP group (*n* = 54; intratibial injection of cancer cells). BCP: bone cancer pain. Data are expressed as the mean ± SEM, and two-way analysis of variance (ANOVA) was used, followed by the Bonferroni test. *P < 0.05 compared with the control group. #*P* < 0.05 compared with the sham group. B. Pathological examination (H&E staining) of ipsilateral bone tissues in the rats in the BCP group. Scale bar = 100 μm, 200 μm

Figure 1A shows the results of the nociception tests 15 days after surgery. Compared with that of the control group, the defense time in the sham-operated group increased significantly from day 1 after surgery (*P* < 0.05) and returned to the baseline level on day 13. However, in the BCP group, the defense time was prolonged over time, and there were significant differences compared with the sham group from day 5 (*P* < 0.05). Pathological results also demonstrated that the BCP models were successfully established **(**Fig. 1B**).**

### Effects of dezocine on morphine tolerance

**Fig. 2:**
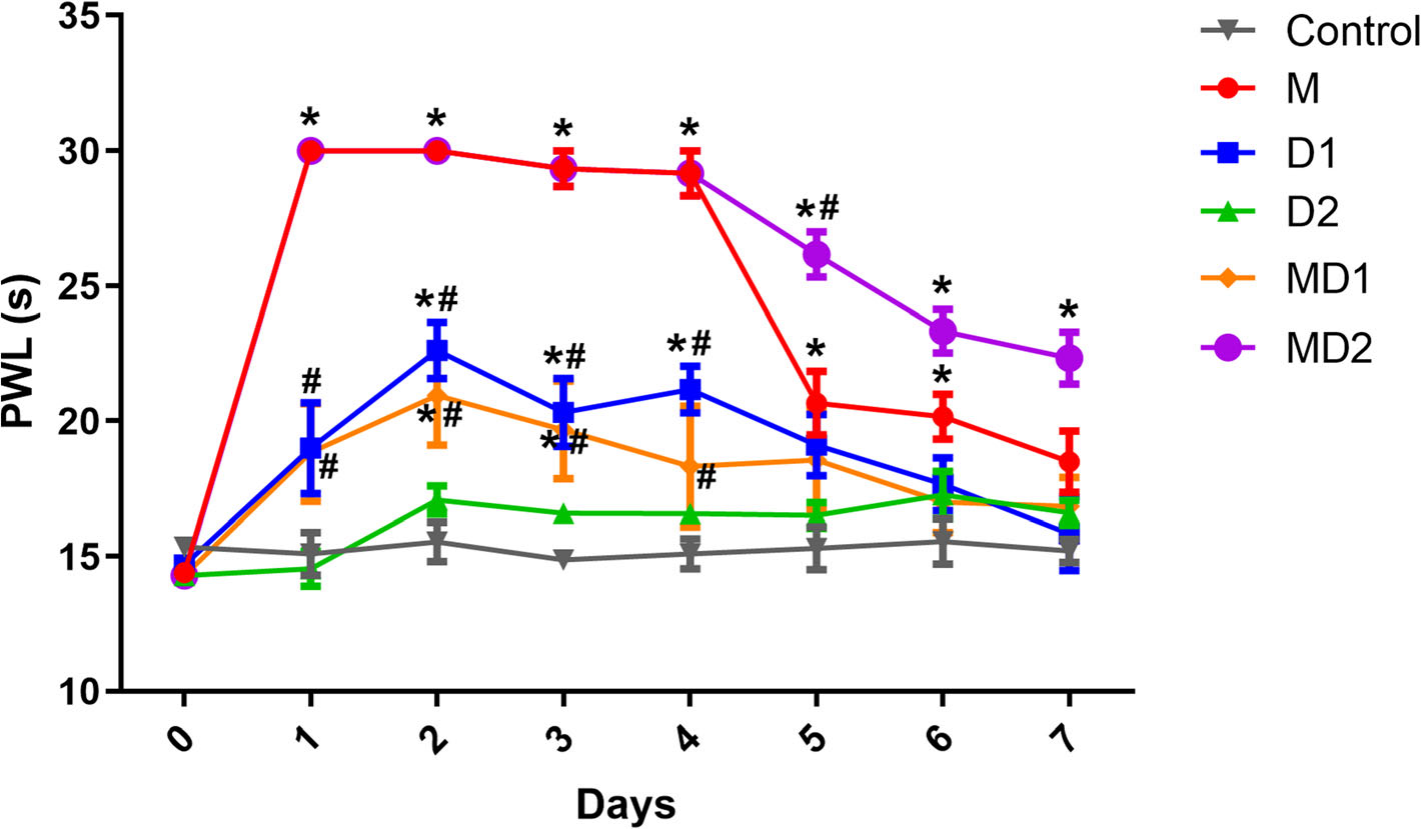
Comparison of the thermal nociceptive thresholds among the groups. Thirty-six rats with BCP were treated with opioids or normal saline twice daily for 7 days. The rats were randomly divided into six groups with different drug regimens (*n* = 6 per group): M group (10 mg/kg morphine), D1 group (10 mg/kg dezocine), D2 group (1 mg/kg dezocine), MD1 group (10 mg/kg morphine + 10 mg/kg dezocine), MD2 group (10 mg/kg morphine + 1 mg/kg dezocine), and control group (1 mL isotonic saline). All rats underwent thermal pain threshold measurements 30 min after drug administration at 7:30 a.m. PWL: paw withdrawal latency. Data are expressed as the mean ± SEM, and two-way analysis of variance (ANOVA) was used, followed by the Bonferroni test for evaluation. *P < 0.05 compared with the control group. #*P* < 0.05 compared with the morphine group

To measure the analgesic response of opioids in the rats with BCP, we recorded PWL under thermal injury over a 7-day period. Before drug administration, the overall mean baseline PWL to thermal injury was 14.6 ± 0.8 s. There were no significant differences in the PWL baseline between different treatment groups. As shown in Fig. 2, both morphine (M group) and the high dose of dezocine (10 mg/kg, D1 group) significantly prolonged the PWL of the rats with BCP. The administration of morphine from days 1 to 6 produced a significant increase in the thermal nociceptive threshold compared with that of the control group (*P* < 0.05). However, there were no significant differences between these two groups on day 7, indicating that morphine tolerance was induced. High-dose dezocine (10 mg/kg) had a weaker analgesic effect and induced analgesic tolerance on day 5 of administration compared with that of the M group. Low-dose dezocine (1 mg/kg, D2 group) had no significant analgesic effect.

When coadministered with morphine, however, low-dose dezocine (MD2 group) significantly enhanced antinociception (*P* < 0.05) and delayed the emergence of morphine tolerance (Fig. 2). In contrast, high-dose dezocine coadministered with morphine (MD1 group) had a negative effect on the analgesic effect of morphine, and there was no significant difference in the thermal nociceptive threshold on day 4 of drug administration compared with that of the control group.

### Changes in opioid receptors in the PAG

**Fig. 3:**
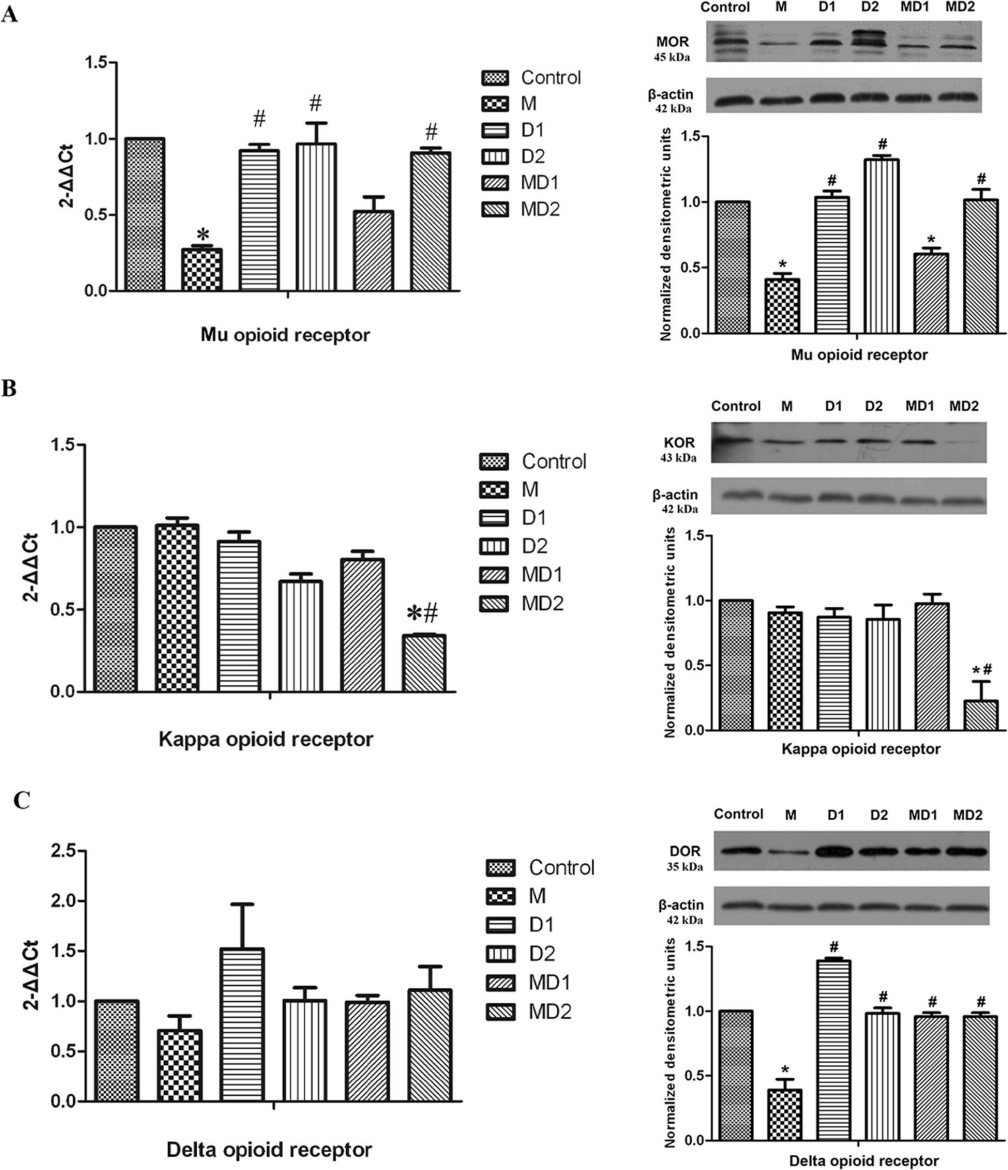
The mRNA and protein levels of opioid receptors in the PAG among the different groups. The groups were as follows (n = 6 per group): control group (1 mL isotonic saline), M group (10 mg/kg morphine), D1 group (10 mg/kg dezocine), D2 group (1 mg/kg dezocine), MD1 group (10 mg/kg morphine + 10 mg/kg dezocine), and MD2 group (10 mg/kg morphine + 1 mg/kg dezocine). Data are expressed as the mean ± SEM. One-way analysis of variance (ANOVA) was used, followed by the Bonferroni test. *P < 0.05 compared with the control group. #*P* < 0.05 compared with the morphine group

The mRNA data were determined by the 2^–ΔΔCT^ method to estimate the expression of opioid receptors in the PAG relative to that in the control group. As shown in Fig. 3A, a 7-day period of morphine administration downregulated the mRNA expression of MOR but did not significantly affect the mRNA expression of KOR (Fig. 3B) or DOR (Fig. 3C) in the PAG. Furthermore, there were no significant changes in opioid receptor expression in the D1 or D2 groups. Dezocine coadministered with morphine (MD1 and MD2 groups) alleviated the morphine-induced downregulation of MOR expression, especially in the MD2 group (Fig. 3A). The MD2 group also showed significantly inhibited KOR expression compared with either the control group or the M group (Fig. 3B).

To investigate the changes in protein levels, we performed western blot analyses of opioid receptors in the PAG of the different groups. In contrast to the findings from the transcriptional analysis, repeated morphine administration resulted in decreased MOR and DOR protein expression in the PAG (Fig. 3A, C). The coadministration of morphine with dezocine appeared to reduce this inhibition of MOR and DOR expression, especially with the low dose of dezocine coadministration. In addition, when compared with that in the other groups, KOR expression was significantly reduced in the MD2 group (Fig. 3B). This result was similar to the observed changes in mRNA levels.

### Changes in opioid receptors in the spinal cord

**Fig. 4:**
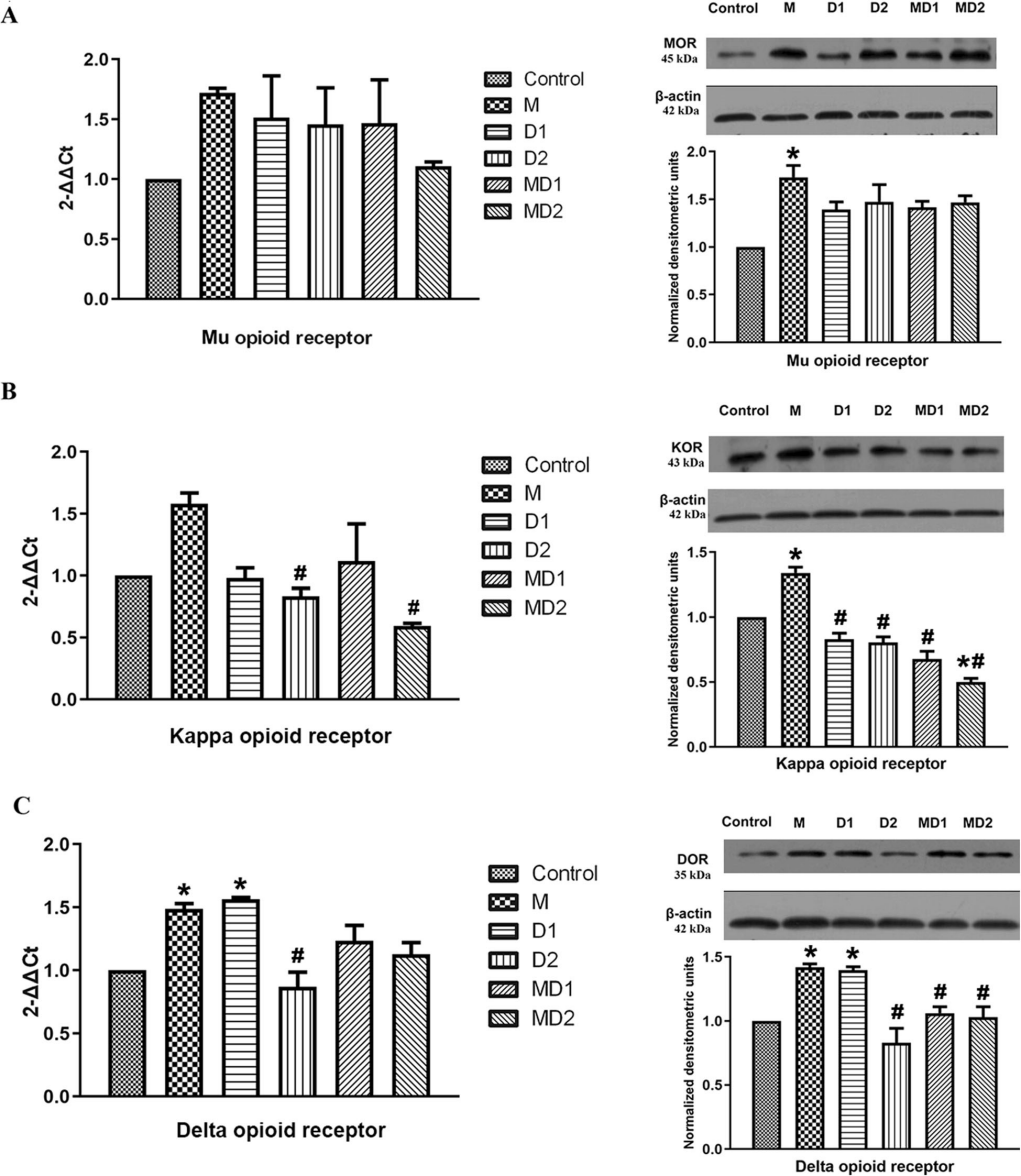
The mRNA and protein levels of opioid receptors in the spinal cords among the different groups. The groups were as follows (n = 6 per group): control group (1 mL isotonic saline), M group (10 mg/kg morphine), D1 group (10 mg/kg dezocine), D2 group (1 mg/kg dezocine), MD1 group (10 mg/kg morphine + 10 mg/kg dezocine), and MD2 group (10 mg/kg morphine + 1 mg/kg dezocine). Data are expressed as the mean ± SEM. One-way analysis of variance (ANOVA) was used, followed by the Bonferroni test. *P < 0.05 compared with the control group. #*P* < 0.05 compared with the morphine group

At the mRNA and protein levels, we observed that a 7-day period of morphine administration upregulated the expression of MOR, KOR and DOR in the spinal cord (Fig. 4). Dezocine seemed to inhibit this phenomenon. Dezocine coadministered with morphine alleviated morphine-induced activation of these opioid receptors. The MD2 group also showed significantly inhibited KOR expression in the spinal cord, similar to the PAG (Fig. 4B).

## Discussion

The present study demonstrated that the analgesic effect of morphine was significantly decreased after 4 days of administration, indicating that tolerance was successfully induced after repeated morphine administration in the BCP rat model. We selected the doses of dezocine according to previous studies [5, 6, 18]. Low-dose dezocine delayed or even prevented morphine tolerance without reducing the analgesic effect of morphine. Opioid receptor expression in the PAG and spinal cord showed different changes in different groups after treatment with morphine alone or in combination with dezocine. The most significant change was that low-dose dezocine coadministered with morphine significantly reversed morphine-induced downregulation of the expression of MORs and DORs in the PAG and upregulation of the expression of KORs and DORs in the spinal cord. Moreover, low-dose dezocine coadministered with morphine significantly inhibited KOR expression in both the PAG and spinal cord.

Pain is the main symptom in 75% of patients with advanced cancer, and bone cancer pain is the most common type of pain [19]. Bone cancer pain is common in bone metastases of some primary tumors, such as lung, breast or prostate cancer [20, 21]. It is often difficult to control pain symptoms with opioids only [22]. The rat model of bone cancer pain can simulate some of the clinical manifestations of cancer pain patients, such as bone destruction and nerve compression [23], and is therefore used in research on the treatment of cancer pain. In the present study, rats in the BCP group showed a significantly prolonged defense time from day 5 compared with rats in the control group, suggesting that a rat model of bone cancer pain had been successfully developed. The occurrence of bone cancer pain involves multiple pathophysiological mechanisms, and satisfactory analgesic results are often difficult to achieve with a particular drug [24, 25]. In recent years, multimodal analgesia has been widely accepted and used in the treatment of bone cancer pain [13]. Although dezocine is often clinically combined with morphine to manage pain, their clinical effect is still controversial [5, 26]. There have been few recent studies on dezocine investigating its efficacy for the treatment of cancer pain.

Dezocine, as a representative opioid receptor agonist-antagonist or partial agonist, has a similar structure to pentazocine. It was previously believed that dezocine is a partial agonist of μ receptors and a full agonist of κ receptors, having a greater affinity for μ and κ receptors than morphine [27–29], while its agonist effect is weaker than that of morphine [30]. We found that dezocine at the same dosage had weaker antinociceptive effects than morphine, which is consistent with theoretical speculation [31]. The combination with low-dose dezocine delayed the tolerance induced by morphine, which is similar to the results in a clinical study that found that low-dose dezocine may enhance the analgesic effect of morphine [5].

However, long-term use of morphine can lead to tolerance via complex mechanisms. Our study found that the expression of MORs decreased in the PAG but increased in the spinal cord after morphine treatment. Morphine exerts its effect mainly by acting on MOR, but the role of MOR in morphine tolerance formation is still controversial. The upregulated expression, downregulated expression, desensitization and internalization of MOR are all possibly related to morphine tolerance [32–34]. Dezocine combined with morphine could have a pronounced effect in the PAG but barely showed effects in the spinal cord on the regulation of MORs. The changes in MORs may be only partially responsible for the effect of morphine tolerance, and KOR and DOR could also play a role in this process. We found downregulated expression of KORs in both the PAG and spinal cord after cotreatment with a low dose of dezocine.

It was previously believed that dezocine is a full agonist of KOR and exerts its analgesic effect by activating KOR. However, recent studies have indicated that dezocine is actually an antagonist of KOR [27, 35]. KOR is expressed in the peripheral nerves, dorsal root ganglia, spinal cord, and supraspinal regions and is closely related to pain regulation. KOR plays an important role in the analgesic effects and tolerance of opioids. Rats tolerant to KOR agonists required increased doses of morphine to achieve the analgesic effect [36]. The analgesic effect of high doses of morphine in juvenile rats tolerant to the analgesic effect of morphine was mediated by KOR [37]. In the present study, KOR mRNA and protein expression was lowest in the groups, and morphine tolerance developed relatively late in the low-dose dezocine plus morphine group, suggesting that low-dose dezocine may delay the development of morphine tolerance. Thus, dezocine may antagonize KOR and reduce tolerance to morphine when administered in combination treatments.

Furthermore, a study has shown that in healthy volunteers, 0.15 mg/kg dezocine alone has a slightly better analgesic effect than morphine. Studies have shown that when the number of ORs was reduced, partial agonists of ORs were more affected than full agonists [38, 39]. In the bone cancer pain model, the levels of MOR in pain-related parts, such as the spinal cord, dorsal root ganglia and periaqueductal gray matter, declined [40, 41], probably because of the sustained release of endogenous opioid peptides in the brain, which results in the activation, phosphorylation, and dysregulation of endogenous production of these molecules and therefore a decrease in MOR levels [42]. Therefore, the analgesic effect of dezocine in bone cancer pain may be weaker than in that in healthy volunteers or acute pain such as postoperative pain. Consequently, our results were reasonable compared with former studies.

Thus, if dezocine is truly a KOR antagonist and a partial agonist of MOR, the results of this study are consistent with the theoretically predicted pharmacological effects. However, controversy still exists over the role of various ORs in morphine tolerance. Further studies are required to investigate the effects of dezocine on KOR and DOR and their interactions with MOR in other regions of the brain. Due to the limited conditions, we did not examine central and peripheral OR affinities, and further studies will be required to address this issue.

In conclusion, the combination of low-dose dezocine with morphine may prevent or delay the development of morphine tolerance and would not decrease the analgesic effect of morphine. The upregulation of MOR and DOR expression in the PAG and downregulation of KOR expression in the PAG and spinal cord may be part of the mechanisms. However, further studies will be required to investigate the efficacy and mechanisms of different doses of dezocine on morphine tolerance.

## Data Availability

The datasets of the current study are available from the corresponding author on reasonable request.
